# Recent Advances towards Validating Efficacy and Safety of African Traditional Medicines

**DOI:** 10.1155/2014/260567

**Published:** 2014-01-16

**Authors:** John R. S. Tabuti, Imed Eddine Hassen, Umar Usman Pateh, Mohamad Fawzi Mahomoodally

**Affiliations:** ^1^Makerere University College of Agricultural and Environmental Sciences, P.O. Box 7062, Kampala, Uganda; ^2^Laboratoire des Méthodes et Techniques d'Analyse (LMTA), Institut National de Recherche et d'Analyse Physico-Chimique (INRAP), Biotechpole de Sidi Thabet, 2020 Sidi Thabet, Tunisia; ^3^Department of Pharmaceutical and Medicinal Chemistry, Faculty of Pharmaceutical Sciences, Ahmadu Bello University, Zaria 1044, Nigeria; ^4^Department of Health Sciences, Faculty of Science, University of Mauritius, 230 Réduit, Mauritius

Traditional medicine (TM) has a significant role to play in the African health care system for two main reasons. The first one is the inadequate access to allopathic medicines and western forms of treatments. The majority of people in Africa cannot afford access to modern medical care either because it is too costly or because there are no medical service providers. Second, there is a lack of effective modern medical treatment for some ailments such as malaria or HIV/AIDS, which, although global in distribution, disproportionately affect Africa more than other areas in the world. It is generally believed that African TMs if revisited and meshed with recent developments in science could provide substantial benefits that could help give a boost to the African health care system and by doing so alleviate sufferings and livelihood of people. It is also believed that by rediscovering and adding value to the ancient wisdoms it will also empower people at the grass-root level and help patients as well as scientists find solutions to mitigate, *inter alia*, the global impact of diseases.

The last few decades have witnessed a growing number of documentations concerning materials used in traditional treatments in Africa. Nonetheless, there is still a paucity of clinical evidence to show that TMs are clinically effective and can be administered to patients safely. Without this information, users of TMs in Africa and elsewhere remain skeptical about the value of some therapies. Additionally, this denies people the freedom to choose complementary and alternative medicines that are potentially less costly and more accessible.

Another matter concerning TMs is the need to understand the clinical safety of these therapies. It is believed that the correct method of studying the effectiveness and potential of TMs should be solely based on scientific principles. If the remedies and practices applied by TMs are efficacious, they should be documented properly and their use should be supported and extended to other fields of practice. On the other hand, if they are found to be clinically ineffective or harmful, their use should be prohibited. One way to establish whether they are effective or not is to embark on robust randomized, double-blind controlled clinical studies. To this effect, there is an ever-pressing need to apply the principles of evidence-based medicine to African TMs, particularly herbal medicines, such that those therapeutic medicinal regimens which prove to be potent and safe become part of conventional medicine and those which fail to meet this standard become obsolete. For these reasons, updated information about efficacy and safety of TM is urgently required. Additionally, the documentation of medicinal uses of African plants and traditional systems is becoming an urgent issue because of the rapid loss of the natural habitats of some of these plants due to anthropogenic activities and also due to an erosion of valuable traditional knowledge.

For the present special issue, original research and review articles that discuss aspects of efficacy and safety of African TMs have been considered. In this special issue, recent findings on evaluations of efficacies of TM from Africa for the management of obesity, stress, antioxidant activities, anticancer properties, and skin pigmentation are collated in 6 original research articles and an overview of priority medicinal plants and fungi in 3 key review articles.

The antiobesity effects of *Nitraria retusa* ethanol extract (NRE) in 3T3-L1 cells and in high fat diet-induced obesity in mice was studied by F. Z. Kalai et al. It was found that NRE administration significantly decreased body weight gain, fat pad weight, serum glucose, and lipid levels in high fat diet-induced obese mice. The mRNA expression results showed an enhancement of the expression of genes related to liver metabolism. It was suggested that NRE treatment had a protective or controlling effect against a high fat diet-induced obesity in C57B6J/L mice through the regulation of expression of genes involved in lipolysis and lipogenesis and thus the enhancement of the lipid metabolism in liver.

M. Ben Othman et al. investigated the antistress properties of *Cymbopogon schoenanthus* (CSEE), growing wild in the southern part of Tunisia and used in local folk medicine as diuretic, antispasmodic, rheumatism, anorexia, and digestive disorders including food poisoning. They demonstrated that pretreatment of SH-SY5Y cells with CSEE significantly inversed H_2_O_2_-induced neurotoxicity. Moreover, CSEE treatments significantly reversed heat shock protein expression in heat-stressed HSP47-transformed cells and mRNA expression of HSP27 and HSP90 in H_2_O_2_-treated SH-SY5Y. They concluded that the effect of CSEE on animal behavior was concordant with a significant regulation of blood serum corticosterone and cerebral cortex levels of catecholamine.

The efficacy of treatment of knee osteoarthritis using Shea nut oil (an extract from the indigenous African *Vitellaria paradoxa* tree) was validated by S.-p. Chen et al. The main findings showed significant change between the baseline and post-16-week testing, both in terms of morphological changes and muscle activity. Pain significantly reduced by the 16th week. Indeed, after sufficient dosage and intervention, the effects of Shea nut Oil include decreased inflammation, increased collagen, amelioration of pain, and improved muscle function. These results indicate that Shea nut oil extract can be used as a complementary option to improve the symptoms and function in relation to knee osteoarthritis.

M. Boulaaba et al. studied the phenolic contents, antioxidant activities, and anticancer effects of *Arthrocnemum indicum*,   a traditional medicinal halophyte used in the treatment of poisonous snakebites and scorpion stings in Tunisia. The antiproliferative effect as compared to the control was characterized by substantial total polyphenol content and high antioxidant activity. DAPI staining revealed that these extracts decrease DNA synthesis and reduce the proliferation of Caco-2 cells which are stopped at the G_2_/M phase. The changes in the cell cycle-associated proteins correlate with the changes in cell cycle distribution. Eight phenolic compounds were also identified. In conclusion, *A. indicum *was found to possess interesting antioxidant capacities associated with a significant anti-proliferative effect explained by a cell cycle blocking at the G_2_/M phase.

The melanogenesis regulatory effect of argan oil (*Argania spinosa*) was evaluated by M. O. Villarel et al. using B16 murine melanoma cells. The oil treatment was reported to cause MITF phosphorylation which subsequently inhibited the transcription of melanogenic enzymes. The inhibitory effect of argan on melanin biosynthesis was attributed to tocopherols as well as the synergistic effect of its components. The results of this study tend to provide the scientific basis for the traditionally established benefits of argan oil and present its therapeutic potential against hyperpigmentation disorders.

M.-S. Wu et al. revealed the cytotoxic effect of the flavonoid fisetin (FIS) on human COLO205 colon cancer cells in the presence and absence of the HSP90 inhibitors, geldanamycin (GA), and radicicol (RAD). A reduction in p53 protein with increased ubiquitin-tagged proteins was observed in COLO205 cells treated with FIS. Furthermore, GA and RAD reduced the stability of the p53 protein in COLO205 cells under FIS stimulation. The evidence supports HSP90 inhibitors possibly sensitizing human colon cancer cells to FIS-induced apoptosis and treating colon cancer by combining HSP90 inhibitors with FIS deserves further *in vivo* study.

M. F. Mahomoodally has provided an updated overview of 10 promising medicinal plants from the African biodiversity which have short- as well as long-term potential to be developed as into phytopharmaceuticals to treat and/or manage a panoply of infectious and chronic conditions. On their part H. El Enshasy et al. have summarized currently available information related to the nutritional and medicinal value of African and Middle Eastern macrofungi and highlighted their application in complementary folk medicine in this part of the world. Lastly, S. Z. Moghadamtousi et al. reviewed information and findings concerning current knowledge on the biological activities, pharmacological properties, toxicity, and chemical constituents of the eastern Nigerian species—*Loranthus micranthus*, which has been widely used in Nigeria and South Africa as ethnomedicine for treatment of hypertension, diabetes, and schizophrenia and as an immune system booster.

Within the structure of enhancing the significance of African TMs, this special issue has highlighted several aspects related to the efficacy and safety of traditional African treatments, particularly medicinal plants, with a focus on promoting rational utilization for better health care. Much more research and documentation is still required to achieve this ambitious goal.

*John R. S. Tabuti* *John R. S. Tabuti* *Imed Eddine Hassen* *Imed Eddine Hassen* *Umar Usman Pateh* *Umar Usman Pateh* *Mohamad Fawzi Mahomoodally* *Mohamad Fawzi Mahomoodally*

